# In silico prediction of novel therapeutic targets using gene–disease association data

**DOI:** 10.1186/s12967-017-1285-6

**Published:** 2017-08-29

**Authors:** Enrico Ferrero, Ian Dunham, Philippe Sanseau

**Affiliations:** 1 Computational Biology and Stats, Target Sciences, GSK Medicines Research Centre, Gunnels Wood Road, Stevenage, SG1 2NY UK; 2 European Molecular Biology Laboratory, European Bioinformatics Institute (EMBL-EBI), Wellcome Genome Campus, Hinxton, Cambridge, CB10 1SD UK; 3 Open Targets, Wellcome Genome Campus, Hinxton, Cambridge, CB10 1SD UK

**Keywords:** Drug discovery, Target discovery, Gene–disease associations, Machine learning, Data mining

## Abstract

**Background:**

Target identification and validation is a pressing challenge in the pharmaceutical industry, with many of the programmes that fail for efficacy reasons showing poor association between the drug target and the disease. Computational prediction of successful targets could have a considerable impact on attrition rates in the drug discovery pipeline by significantly reducing the initial search space. Here, we explore whether gene–disease association data from the Open Targets platform is sufficient to predict therapeutic targets that are actively being pursued by pharmaceutical companies or are already on the market.

**Methods:**

To test our hypothesis, we train four different classifiers (a random forest, a support vector machine, a neural network and a gradient boosting machine) on partially labelled data and evaluate their performance using nested cross-validation and testing on an independent set. We then select the best performing model and use it to make predictions on more than 15,000 genes. Finally, we validate our predictions by mining the scientific literature for proposed therapeutic targets.

**Results:**

We observe that the data types with the best predictive power are animal models showing a disease-relevant phenotype, differential expression in diseased tissue and genetic association with the disease under investigation. On a test set, the neural network classifier achieves over 71% accuracy with an AUC of 0.76 when predicting therapeutic targets in a semi-supervised learning setting. We use this model to gain insights into current and failed programmes and to predict 1431 novel targets, of which a highly significant proportion has been independently proposed in the literature.

**Conclusions:**

Our in silico approach shows that data linking genes and diseases is sufficient to predict novel therapeutic targets effectively and confirms that this type of evidence is essential for formulating or strengthening hypotheses in the target discovery process. Ultimately, more rapid and automated target prioritisation holds the potential to reduce both the costs and the development times associated with bringing new medicines to patients.

**Electronic supplementary material:**

The online version of this article (doi:10.1186/s12967-017-1285-6) contains supplementary material, which is available to authorized users.

## Background

In drug discovery, programme failures at late stages of development such as clinical phases are extremely costly [1]. In the majority of cases, it appears that lack of efficacy is often the primary cause of this attrition [2, 3]. Efficacy failures, in turn, are most often due to a poor linkage between the therapeutic drug target and the disease of interest or the lack of a well validated animal model of the disease [4]. Hence, the selection of the right target for the right disease in early discovery phases is a key decision to maximise the chances of success in the clinic and ensure a sustainable business in the longer term [5].

Many different sources of evidence linking potential therapeutic targets to diseases can be used in the target selection process. However, it is currently unclear what data type(s) are more relevant or appropriate to use when picking new drug targets. Recent reports highlighted that human genetics evidence providing a clear link between the putative target and the disease can have a quantifiable impact on the clinical success rates of new drugs [6, 7]. This is also supported by the finding that phase II projects where a genetic link has been established are almost twice as likely to be active or successful [4]. While genetic associations from large-scale genome-wide association studies (GWAS) are already contributing to the advancement of therapeutic targets in early discovery [8, 9], other data types have not been systematically compared or taken into consideration so far.

Open Targets is a public–private partnership that aims to collect all data that can be used to link genes and diseases, with the ultimate objective of providing evidence on the validation of potential therapeutic targets in one or more disease areas [10]. This is implemented through an informatics platform that integrates multiple pieces of evidence connecting genes and diseases, including genetics (both germline and somatic mutations), gene expression, literature, pathway and drug data [11].

Considering that the drug discovery process is costly and failure-prone, methods that can effectively predict or prioritise which targets to go after to treat or cure major diseases would be welcomed by the scientific community. Our aim is to leverage the notion that clear target–disease associations appear to be related with the success of pharmaceutical programmes; specifically, we are interested in addressing whether human gene products can work as therapeutic targets based on their disease association profile.

Machine learning is emerging as a specialised branch of statistics and computer science that can lead to powerful insights in a number of different domains and contexts [12]. Here, we asked whether a predictive modelling strategy could be applied to identify therapeutic targets: Is it possible to discriminate between current drug targets in the pharmaceutical industry and other genes using a machine learning approach? Is there a set of disease association features that can be used to define drug targets? If so, can we use this information to predict novel targets?

In this study, we take advantage of the Open Targets platform using a semi-supervised approach on positive and unlabelled data to assess whether the disease association evidence that it contains can be used to make de novo predictions of potential therapeutic targets. We test the information content of five types of evidence connecting genes with diseases (pathways, animal models, somatic and germline genetics and RNA expression) and evaluate four different classification algorithms for predicting new drug targets.

## Methods

### Software

All data processing and analysis was performed using R 3.3.0 [13]. The mlr package [14] was used to build the classifiers, test the models and perform the predictions. The underlying packages for building the individual models were rpart [15], randomForest [16], nnet [17], e1071 [18] and gbm [19]. Other packages used for data processing and visualisation were biomaRt [20], jsonlite [21], ggplot2 [22], Rtsne [23] and Vennerable [24]. Release 84 of Ensembl [25] was used for gene annotation. SciBite DocStore [26] was used for text mining the MEDLINE database. All code was versioned using Git and is available at https://github.com/enricoferrero/TargetPred.

### Data processing

Observations and features were collected from the Open Targets platform [11]. It utilises seven distinct data types to represent associations between genes and diseases: affected_pathway (the gene is part of a pathway that is affected in disease), animal_model (animal model with a gene knockout that manifests in phenotype concordant with human disease), genetic_association (germline mutation in the gene associated with the disease), known_drug (existing drug that engages the target and is used to treat the disease), literature (association between gene and disease identified through text mining of the scientific literature), rna_expression (significant gene expression change in disease) and somatic_mutation (somatic mutation in the gene associated with the disease, typically cancer). Each of these data types is composed of one or more data sources. For instance, genetic_association contains germline mutation evidence from the GWAS catalog [27], UniProt [28] and EVA [29] while the somatic mutation data type is a collection of cancer-related mutation data from COSMIC [30] and EVA [29]. For each gene–disease combination, the platform provides association scores for each of the data types as well as an overall association score calculated using the sum of the harmonic progression of each data type score [11]. Individual diseases are represented in the platform according to the Experimental Factor Ontology [31] and grouped in different therapeutic areas via the ontology relationships [11]. The platform stores both direct and indirect associations between genes and diseases, with the indirect associations representing associations between genes and parent terms in the ontology [11].

Four main steps were taken to reshape the raw data from the Open Targets platform into the input format for the machine learning algorithms:

1. The JSON file containing all Open Targets gene–disease associations (2016 Apr version) was downloaded from the platform download page (https://www.targetvalidation.org/downloads/data) and imported into R in tabular format.
2. Five data types were used to build the input data matrix: affected_pathway, animal_model, genetic_association, rna_expression and somatic_mutation. The column corresponding to the known_drug data type was removed from the input data matrix because it is essentially equivalent to what we aim to predict (i.e.: is this gene a drug target?). Similarly, the literature data type was also removed as it was likely to be heavily biased towards well known, validated target–indication pairs and, in addition, we planned to use data from the scientific literature as a means to validate our approach. The animal_model data type was filtered to eliminate lower confidence associations below a 0.4 threshold. Finally, to avoid artificially increasing scores or counting evidence more than once, we removed all indirect associations.
3. For each data type, a single pan-disease score was computed per gene by calculating the mean score across all associated diseases. This ensures that the resulting matrix has a single row per gene and lets the classifier(s) make predictions on individual targets, rather than target–indication pairs.
4. For each gene in the input data matrix obtained from the Open Targets platform, a label (or outcome variable) specifying whether the gene was pursued as a drug target was then added according to the Informa Pharmaprojects data [32]. While the repertoire of target–indication pairs in the Open Targets platform and the Pharmaprojects database is likely to differ, we integrated the two resources at the gene level, without taking into consideration disease association differences that may exist in the two resources. A gene was labelled as a target if it was found in one of the following Pharmaprojects categories: Preclinical, Clinical Trial, Phase I Clinical Trial, Phase II Clinical Trial, Phase III Clinical Trial, Pre-registration, Registered, Launched. For targets with programmes across different stages of the drug discovery pipeline, the most advanced stage was considered. All other genes in the Open Targets dataset were labelled as non-targets.

### Model training and testing

All targets were selected along with an equal number of randomly chosen non-targets to generate a working dataset, while the remaining non-targets were kept as a prediction set.

The working dataset was used for unsupervised exploratory data analyses: hierarchical clustering, principal component analysis and t-stochastic neighbour embedding (t-SNE) [33]. The hierarchical clustering analysis was run using Euclidean distance and Ward’s criterion [34]; the t-SNE method was run using a perplexity value of 30 and other default parameters as implemented in the Rtsne package [23]. The working dataset was then randomly split into training and test sets, containing 80 and 20% of the observations, respectively. The training data was used for tuning hyperparameters and for evaluating the performance of four different classifiers: a random forest (RF) [35], a support vector machine (SVM) with a radial kernel [36], a feed-forward neural network (NN) with a single hidden layer [37] and a gradient boosting machine (GBM) [38] using the AdaBoost exponential loss function [39]. In a positive–unlabelled (PU) learning setting, conventional binary classifiers will often suffer from classifier instability, caused by the fact that the unlabelled set—which is effectively treated as the negative set—contains both positive and negative cases [40, 41]. Bootstrap aggregating (bagging) is a common and effective technique that can be used to reduce classifier instability by randomly resampling observations with replacement and then aggregating the results by majority voting [42]. We applied bagging with 100 iterations to the SVM, the NN and the GBM classifiers. RFs already implement the bagging procedure by default [35] so the classifier was not modified.

To ensure that performance estimates were reliable and that the models were not overfitted to the training data, a nested cross-validation strategy was adopted [43, 44]. An inner, fourfold cross-validation loop was used to tune the following hyperparameters of the four classifiers: number of trees and number of features (RF); size and decay (NN); gamma and cost (SVM); number of trees and interaction depth (GBM). An outer, fourfold cross-validation loop was then used for estimating the performance of the classifiers. The performance of the four models was then evaluated on the test set. Based on these benchmarks, the NN model was selected and used to make predictions on the remaining set of non-targets (prediction set) using a probability threshold of 0.9.

### Text mining

The SciBite DocStore API [26] was utilised to identify instances of “Gene/Protein AND Target” in titles and abstracts stored in the MEDLINE database, where “Gene/Protein” stands for all known genes and proteins and “Target” is a concept describing a therapeutic target. Results were retrieved in a tab-delimited format and processed so that gene symbols could be mapped to Ensembl gene IDs. A two-sided Fisher’s exact test was then used to assess the significance of the overlap between predictions and text mining hits using the total number of protein-coding genes in the human genome as the universe size.

## Results

**Fig. 1:**
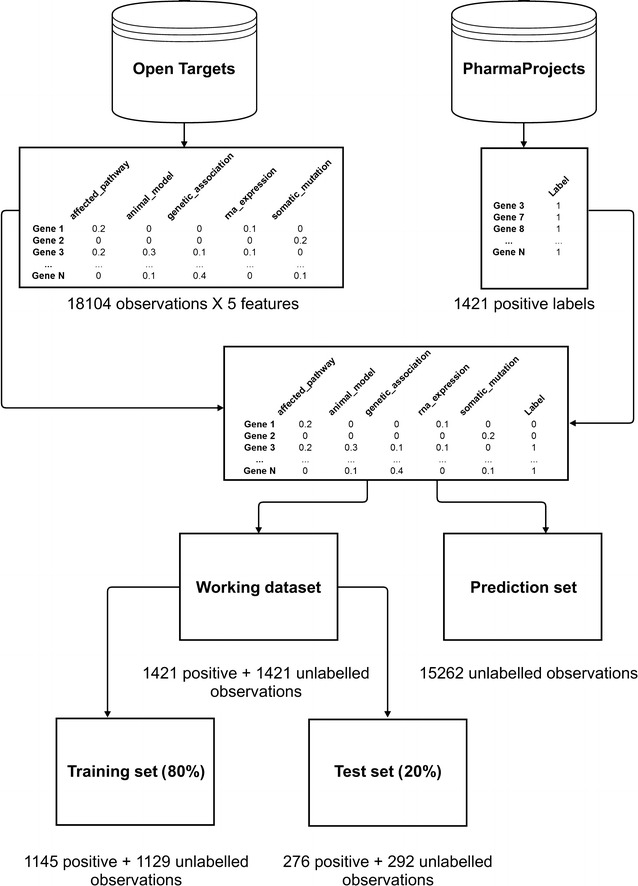
Framework for prediction of therapeutic targets based on disease association data. Observations and features were gathered from Open Targets while the labels were collected from Informa Pharmaprojects. The resulting data matrix was split into a prediction set and a working dataset. The former was kept aside and used to perform predictions once the model was established. The latter, containing both positive and unlabelled observations, was further split into training and test sets to train the classifiers and evaluate their performance

To assess whether information providing evidence of association between genes and diseases could predict successful drug targets, we set out to train and test a number of algorithms on an input matrix built using data from the Open Targets platform [11]. We obtained a matrix with 18,104 genes (observations) and five data types from Open Targets (features) by summarising all the available evidence at the gene level. Observations were labelled as targets or non-targets according to the presence or absence of drugs marketed or in development across the pharmaceutical industry using the Informa Pharmaprojects data [32] (Fig. 1). Information on which gene products are drug targets is available directly in the Open Targets platform though ChEMBL [45]. However, at the time of this analysis, the number of targets with drug annotations available from Pharmaprojects, which also covers early stage announcements, was superior (2105 compared to 625), with the majority of those found in Open Targets also part of the Pharmaprojects collection (389). In addition, using the Pharmaprojects data makes it possible to easily discriminate between the drug development phases and filter out targets with failed or abandoned programmes when generating the positive set.

One issue that we faced is that our data is only partially labelled. We have a number of genes that are currently targets of drugs that are marketed or under active development but, for the majority of genes, we don’t know with certainty whether they are definitely non-targets or might become targets in the future, as our understanding of diseases progresses and drug discovery technologies advance. Semi-supervised learning methods tackle exactly this type of settings, where a mixture of labelled and unlabelled data exists [46]. Specifically, a scenario where all labelled observations are positives is known as PU learning, or learning from positive and unlabelled data [47]. We created a positive set using all known drug targets, with the remaining protein-coding genes represented in Open Targets falling in a bucket of non-targets. The assumption is that this unlabelled set contains both negatives—genes that are never going to be drug targets because of efficacy, safety or tractability reasons—and future positives, genes that are currently not being actively pursued as therapeutic targets, but will become so in the future. From an algorithmic perspective, we simply treated the unlabelled data as the negative set [48], allowing us to utilise supervised learning methods within a semi-supervised setting.

A balanced working dataset was generated containing all positive cases (1421) and an equal number of randomly sampled unlabelled cases (2842 in total). The remaining 15,262 observations were set aside to form a prediction set to be used for the actual predictions once a definite model had been established (Fig. 1).

### Targets and non-targets appear as distinct on a two-dimensional space

We carried out an exploratory analysis on the working dataset using unsupervised methods to understand whether targets and non-targets had different characteristics.

As expected, hierarchical clustering of the data revealed a very sparse matrix, with most values close to zero and virtually no genes showing high scores across multiple data types (Additional file 1: Figure S1A). While some smaller clusters predominantly composed of targets or non-targets could be identified, overall the data structure did not appear to relate to the assigned labels.

Similarly, a principal component analysis showed substantial overlap of targets and non-targets when plotting pairwise combinations of the first three principal components, that altogether accounted for 66.1% of the total variance in the data (Additional file 1: Figure S1B).

**Fig. 2:**
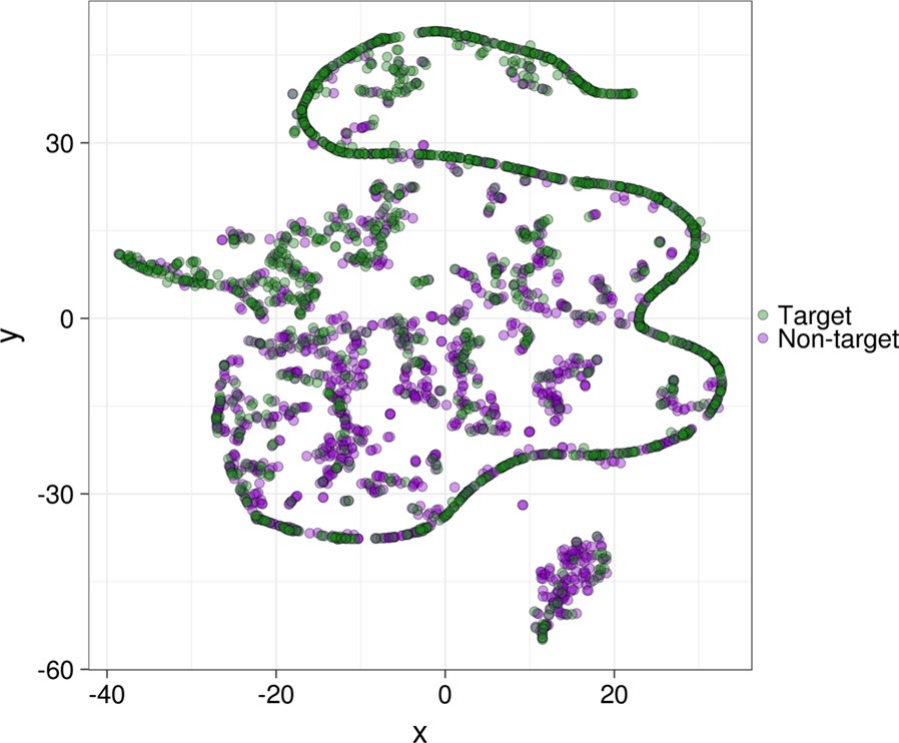
Exploratory data analysis of the working dataset using dimensionality reduction. The t-SNE algorithm for non-linear dimensionality reduction was run a perplexity value of 30 and other default parameters. Each *dot* in the two-dimensional space represents a gene and is coloured according to its label (*green* target, *purple* non-target)

We then asked whether a more sophisticated dimensionality reduction method such as t-SNE [33] could reveal some hidden structure in the data. This resulted in a rather clear separation between targets and non-targets on a two-dimensional space (Fig. 2): most of the data points labelled as targets lie on a curved line and a gradient of targets and non-targets is present along the vertical axis.

This result shows that a distinction between therapeutic targets and other genes exists based on the Open Targets data, thus supporting the notion of a non-linear classification-based approach that discriminates between targets and non-targets using disease-association evidence.

### Assessing feature importance and classification criteria

Considering that the feature space under investigation was small, we set out to investigate the contributions of all five individual data types and their relative importance in the dataset.

In scenarios with several predictors, it is common practice to use feature selection as a means of simplifying model interpretation, reducing training times and to avoid overfitting models to the training data [12, 49]. Many feature selection methods rely on assessing the importance of the different features by calculating how related they are with the response variable. Here, we applied the Chi squared test and the information gain method [50] to our dataset to understand which variables were considered more relevant, without actually filtering out any of the original features.

**Fig. 3:**
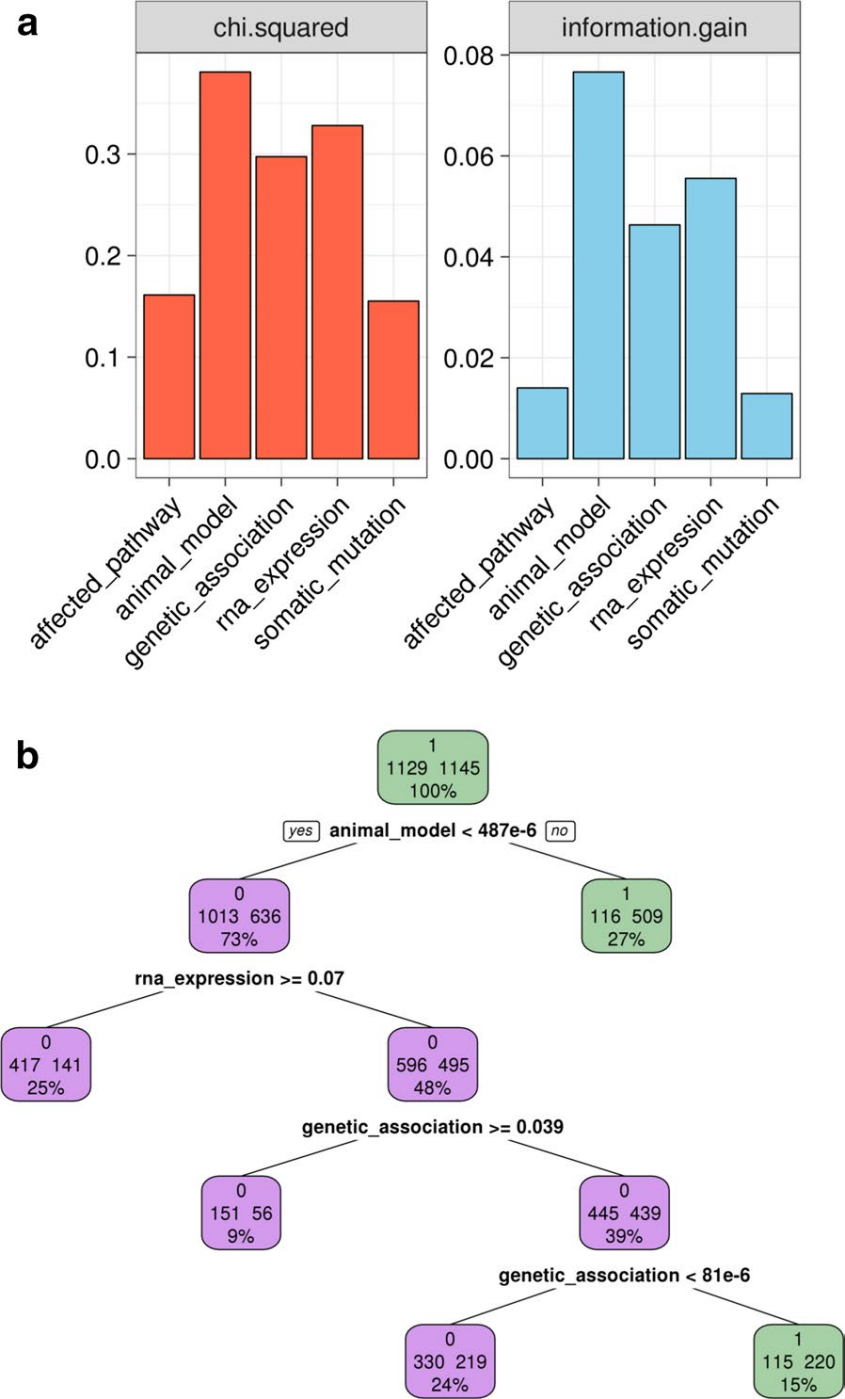
Feature importance and classification criteria. **a** Feature importance according to two independent feature selection methods (*left to right*): Chi squared test and information gain. **b** Decision tree classification criteria: colours represent predicted outcome (*purple* non-target, *green* target). In each node, numbers represent (from *top to bottom*): outcome (0: non-target, 1: target), number of observations in node per class (*left* non-target, *right* target), percentage of observations in node

Regardless of the method utilised, we observed animal model, RNA expression and genetic association showing the highest values and association with the outcome variable (Fig. 3a).

These results suggest that evidence from animal model, gene expression and genetic data can potentially be helpful to define what makes a good therapeutic target. Hence, classifiers built on top of the dataset under investigation are more likely to prioritise this subset of features. Importantly, directionality is not taken into account by these methods so there is no way to know whether it is a higher or a lower score of any of these data types that is more correlated with what we are trying to predict.

Several machine learning algorithms exist for classification tasks, with varying degrees of performance and interpretability [12]. The choice of the right algorithm depends on many factors—often specific to the dataset currently being studied—and ultimately hinges on whether we are more interested in inference (e.g.: what makes a good target?) or prediction (e.g.: which ones are good targets?) [12].

Decision trees are a popular choice for inference since they are easy to interpret without sacrificing too much performance in most scenarios [51]. To test our hypothesis that the features described above would be prioritised by a learning algorithm, we trained a classification tree on a subset of the data—the training set—corresponding to 80% of the observations. We then explored the classification criteria of the model to get insights into the features the algorithm uses to make classification decisions (Fig. 3b).

In line with the feature importance results described above, we found that animal model was the first node in the tree, with RNA expression and genetic association also required for target classification. These findings confirmed the rich information content present in these predictors and suggested that more powerful non-linear classification approaches could indeed achieve satisfactory separation of the two classes.

### Different learning algorithms can predict therapeutic targets with good accuracy

**Fig. 4:**
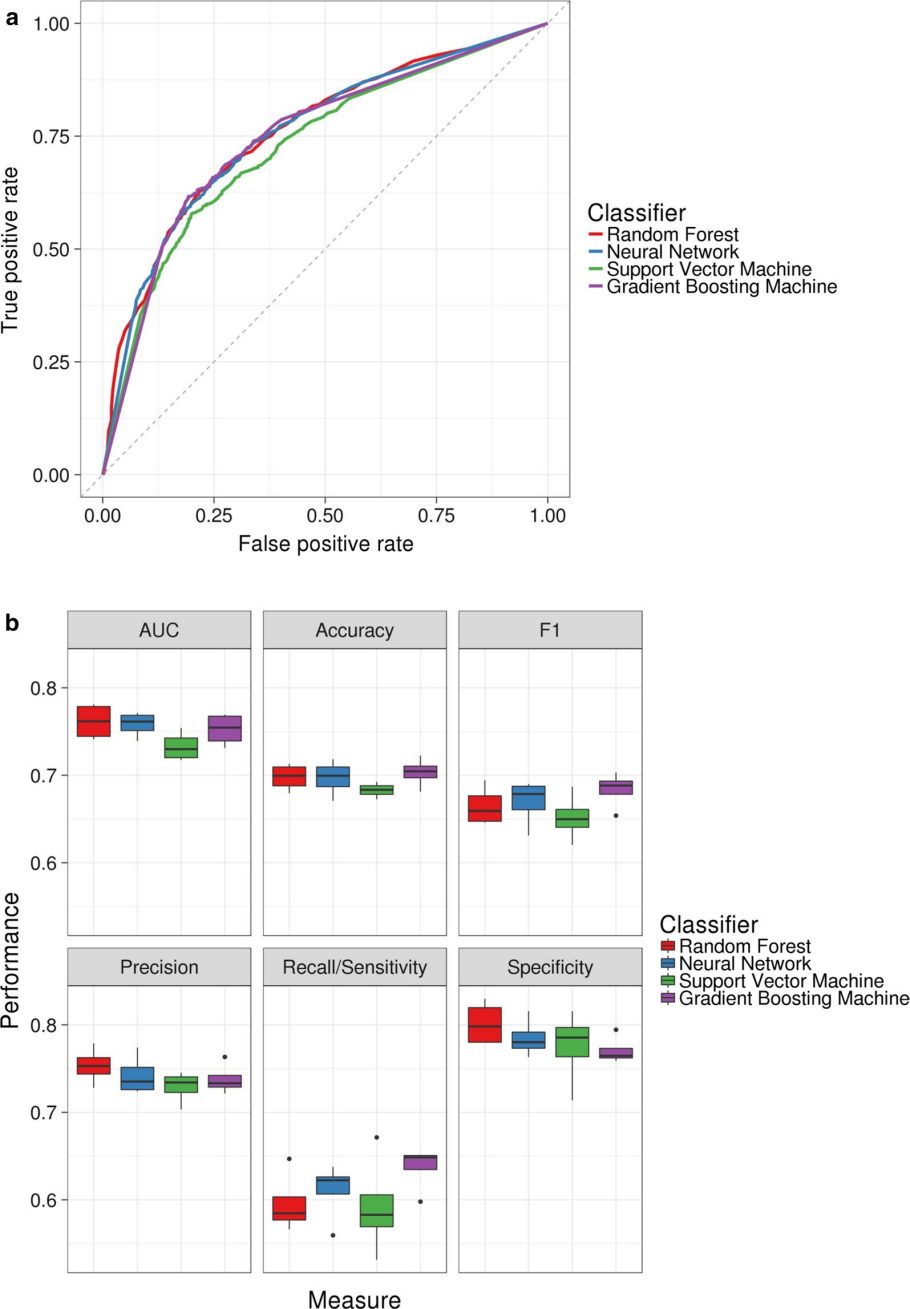
Estimated performance measures of trained classifiers as assessed by nested cross-validation on the training set. **a** Receiver operating characteristic curves. **b** Box plots showing distributions of the following measures for the four algorithms: AUC, accuracy; F1 measure, precision, recall/sensitivity and specificity

We selected four learning algorithms that are generally known to achieve good performance across a number of settings: a RF, a SVM, a NN and a GBM. To account for classifier instability due to the PU learning setting [40, 41], bootstrap aggregating (bagging) with 100 iterations was applied to the SVM, the NN and the GBM classifiers. In order to avoid overfitting during the model tuning and evaluation procedures, we used a nested cross-validation strategy [43, 44] with a fourfold inner loop to tune hyperparameters and a fourfold outer loop to estimate the performance of the algorithms on the training set. Interestingly, we observed the four methods to have broadly similar performance, with no classifier clearly outperforming the others. The receiver operating characteristic (ROC) curves showed substantial similarity at different thresholds of the true positive and false positive rates (Fig. 4a). Of note, since the unlabelled set contains both positives and negatives, the false positive rate (FPR) is overestimated when compared to a standard supervised setting [48]. Another way to benchmark these classifiers is to look at precision–recall curves (Additional file 2: Figure S2A): while the SVM shows slightly lower values, the remaining three classifiers display a decent trade-off between precision and recall.

We then calculated the following standard performance measures for all models: area under the curve (AUC), accuracy, precision, recall/sensitivity, specificity and F1 score (Fig. 4b). Overall, we observed that all algorithms had comparable and satisfactory accuracy, AUC, precision and specificity. The recall/sensitivity was generally found to be somewhat lower, possibly highlighting a limitation in identifying true positives or simply reflecting the fact that a number of the unlabelled observations are actually positives. Of note, the SVM appeared to moderately underperform compared to the other algorithms, while the GBM model displayed a slightly better overall performance during cross-validation. Accordingly, it also achieved the lowest median misclassification error (Additional file 2: Figure S2). We report the mean values of all performance measures for the four classifiers in Table 1.

**Table 1:** Mean training set performance measures for all classifiers estimated by nested cross-validation

Classifier	Misclassification error	Accuracy	AUC	Sensitivity/recall	Specificity	Precision	F1 score
RF	0.302	0.698	0.761	0.596	0.802	0.753	0.665
NN	0.303	0.697	0.758	0.610	0.785	0.742	0.670
SVM	0.317	0.683	0.733	0.592	0.775	0.729	0.652
GBM	0.297	0.703	0.752	0.637	0.771	0.738	0.683

Finally, we explored how consistent the predictions were across models and observed a high degree of overlap. All four algorithms agreed on the classification of the majority of the observations in the training set for both targets (747, 66.4%, Additional file 3: Figure S3A) and non-targets (1149, 75.2%, Additional file 3: Figure S3B).

We have used cross-validation extensively in this study because it provides reliable estimates for the test error rate without having to resort to data outside of the training set [12, 43, 44]. However, to further ensure that we were not overfitting the models to the training data, we evaluated the performances of the four classifiers on an independent test set (corresponding to 20% of the observations in our working dataset) that was not previously fed to the learning algorithms.

We found the performance of all models to be consistent with the nested cross-validation results on the training set, indicating that overfitting did not occur (Table 2). The best performing classifier on the test data was the NN: it achieved an AUC of 0.76 and accuracy above 71%, meaning that less than 29% of the observations were misclassified. The model featured good precision (0.74) and specificity (0.78); on the other hand, the recall/sensitivity and the F1 score were lower, but still well above 60% (0.64 and 0.68, respectively).

**Table 2:** Test set performance measures for all classifiers

Classifier	Misclassification error	Accuracy	AUC	Sensitivity/recall	Specificity	Precision	F1 score
RF	0.290	0.710	0.761	0.645	0.771	0.727	0.683
NN	0.287	0.713	0.763	0.638	0.784	0.736	0.683
SVM	0.296	0.704	0.747	0.594	0.808	0.745	0.661
GBM	0.294	0.706	0.750	0.649	0.760	0.719	0.682

### Using a neural network to predict drug targets based on disease association data

Based on the benchmark results, we selected the bagged NN as the classifier with the most balanced overall performance and further explored the results as shown in confusion matrices (Table 3). In line with the performance measures reported above, we found that the NN model was able to identify true positives and true negatives, but also misclassified a proportion of targets as non-targets because of a lower sensitivity.

**Table 3:** Confusion matrices for the neural network model

	Predicted: non-target	Predicted: target	Sum
A. Training set (nested cross-validation)			
Actual: non-target	886	243	1129
Actual: target	446	699	1145
Sum	1332	942	2274
B. Test set			
Actual: non-target	229	63	292
Actual: target	100	176	276
Sum	329	239	568

To ensure that our results were not biased by the random sampling of non-targets, we used a Monte Carlo simulation to select several different subsets of the unlabelled data (n = 10,000) for the training and test sets. At each iteration, we created a new training set by adding the positive class and trained a NN classifier that was then tested on an independent test set (Additional file 4: Figure S4A, B). We found that the performance measures of our classifier were largely unaffected by the random sampling step (mean accuracy = 0.69, standard deviation = 0.02; mean AUC = 0.75, standard deviation = 0.02).

**Fig. 5:**
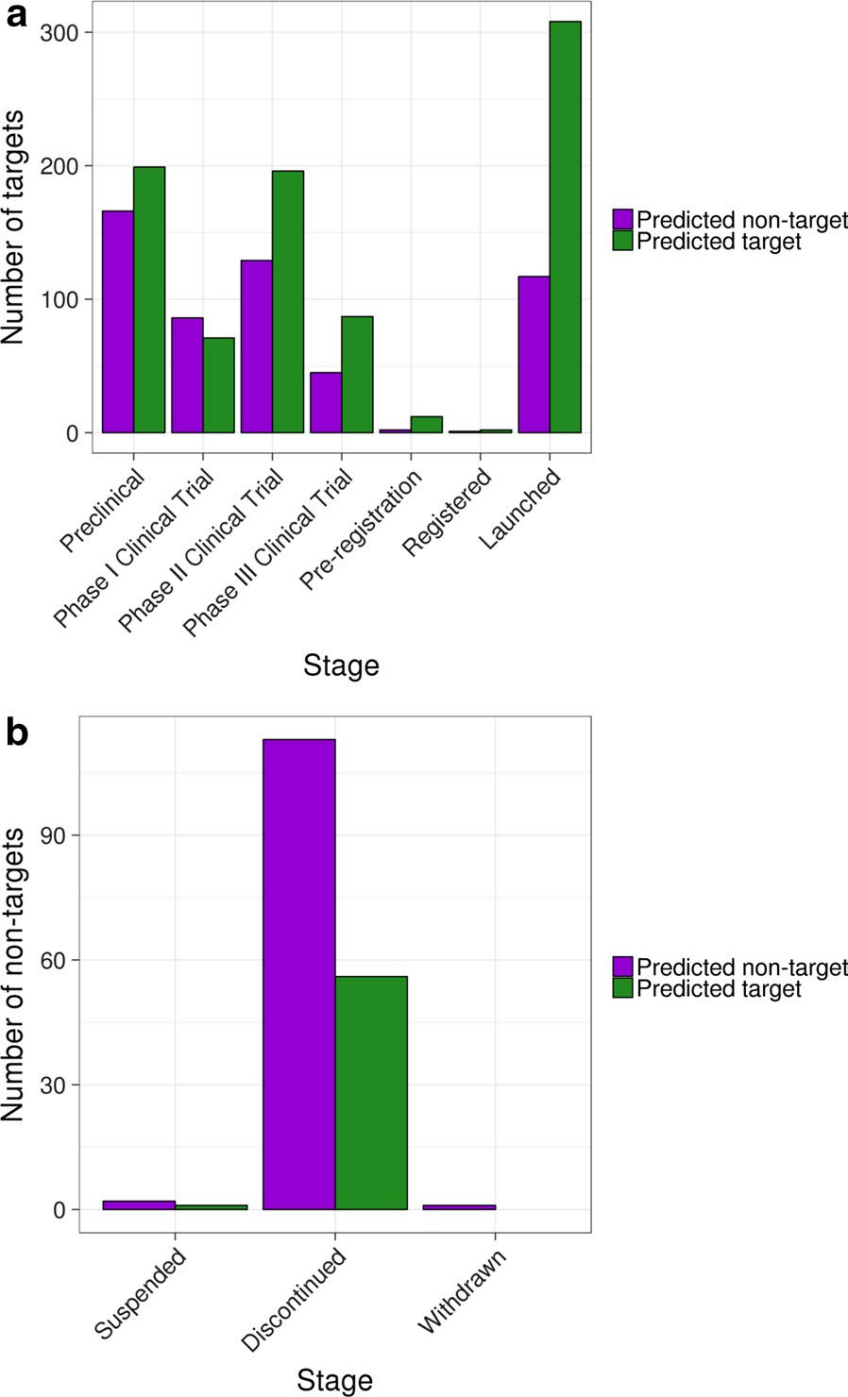
Distribution of targets across different stages of the drug discovery pipeline. **a** Known drug targets predicted as non-targets (*purple*) or targets (*green*) during training and testing. **b** Non-targets predicted as non-targets (*purple*) or targets (*green*) during training, testing and prediction

The 1421 known targets utilised in the training and test set were further explored at this stage. Considering both training and testing, 875 genes (61.6%) were correctly predicted as targets while the remaining 546 (38.4%) were predicted as non-targets (Table 3). We set out to understand whether there was any difference between these two groups based on how advanced the targets were in the drug discovery pipeline (Fig. 5a).

We observed that many of the targets correctly identified as such belonged to drugs currently on the market (308, 35.2%), while the proportion of launched drugs for predicted non-targets was much lower (117, 21.4%). A similar trend was observed for programmes currently in phase II and III clinical trials; conversely, targets in earlier phases were more equally distributed among the two classes. We confirmed this by using logistic regression and found significant differences for the following stages: launched (p = 4.97e−19), pre-registration (p = 0.02), phase III clinical trial (p = 3.30e−4) and phase II clinical trial (p = 2.25e−4). These results suggest that therapeutic targets more advanced in the drug discovery pipeline show clearer differences and thus appear more straightforward to discriminate.

We also asked whether this model was capable of predicting failed targets by examining what we predicted as non-targets and the number of drugs that were suspended or discontinued during development or withdrawn from the market (Fig. 5b). We found that the total number of predicted non-targets belonging to discontinued programmes (113) was significantly higher than the number of predicted targets (56; logistic regression p = 1.74e−5), suggesting that our model is—to some extent—able to discriminate between targets that will or will not fail during development.

Finally, we used the model to make predictions on all the remaining 15,262 unlabelled observations not included in the training or test sets, and ranked them by their probability of being a drug target (Additional file 5: Table S1). By default, all observations with probability higher than 0.5 of being a target are classified as such. In an effort to reduce the number of false positives in our predictions, we applied a more stringent probability cut-off of 0.9, which resulted in 1431 genes being predicted as novel targets according to their disease association profile.

### Literature text mining validates predictions of novel targets

**Fig. 6:**
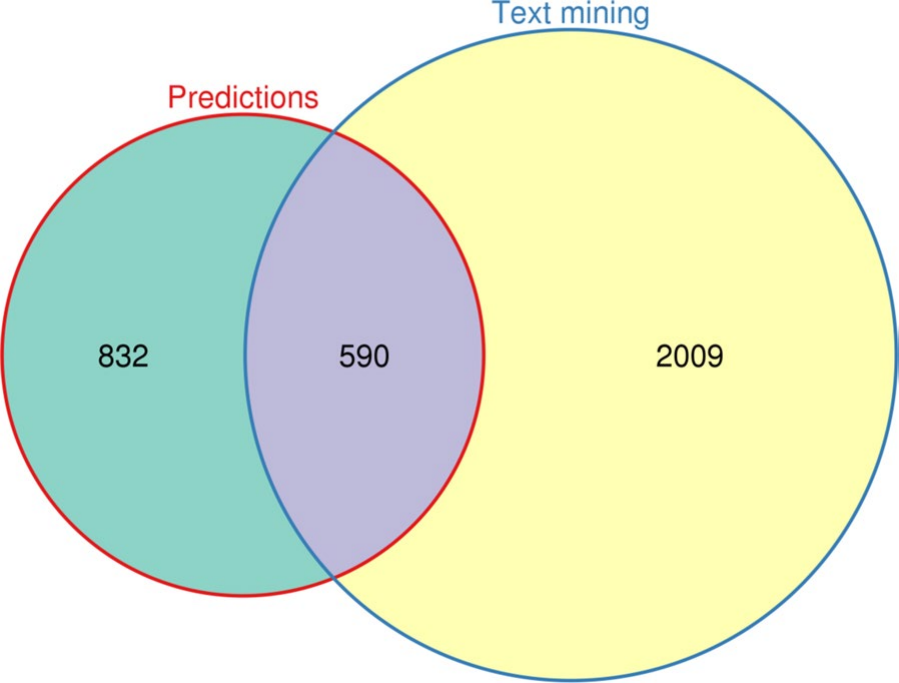
Validation of target predictions using the scientific literature. The Venn diagram shows the overlap between the predicted targets from the NN classifier and those retrieved from the literature through text mining

The purpose of performing cross-validation or using a test set is to assess the performance of a classifier and validate its predictions using previous knowledge. However, an intrinsic limitation of these methods is that the same *type* of data (albeit not the same data) is used for validating the approach. Thus, we utilised the scientific literature as an external source of validation by retrieving suggested drug targets from published articles and checking what proportion of these we were predicting with our model. Specifically, we searched for occurrences of a gene or protein being flagged as a (potential) therapeutic target in titles and abstracts on MEDLINE and found 25,603 such instances, corresponding to 4413 unique genes (Additional file 6: Table S2). From this set, we removed all genes included in the training and test set and calculated the overlap with the NN predictions (Fig. 6). We found 590 genes in common between the two sets, a highly significant proportion as assessed by Fisher’s exact test (p = 5.05e−172, odds ratio = 5.78). To exclude that this result could be due to random chance, we computed 10,000 random permutations and calculated p values and odds ratios using the same statistical test. Neither of these values came close to the results obtained with the original data (Additional file 7: Figure S5A, B). These results serve as an external source of validation of the approach described here and demonstrate that the types of disease-association data used in this study can be predictive of therapeutic targets with good accuracy.

## Discussion

We have presented a machine learning approach that is able to make accurate predictions of therapeutic targets based on the gene–disease association data present in the Open Targets platform, demonstrating that disease association is predictive of the ability of a gene or a protein to work as a drug target. Importantly, our predictions are individual targets, and not target–indication pairs: we predict potential therapeutic targets, regardless of the intended indication. These findings provide the first formal proof that drug targets can be predicted using solely disease association data and strengthen the hypothesis that establishing unambiguous causative links between putative targets and diseases is of paramount importance to maximise the chances of success of drug discovery programmes [4–7]. Notably, an early discovery in silico pipeline able to prioritise candidate targets could lead to decreased failures for efficacy reasons at later stages, potentially resulting in substantial time and cost savings in the drug discovery process [1–4].

The data collected and made available by Open Targets [11] is emerging as a key resource for target identification and validation field work. A number of databases of systematic gene–disease associations existed prior to Open Targets, such as the Comparative Toxicogenomics Database [52], DisGeNET [53] and DISEASES [54]. However, these initiatives focus on evidence that is either manually curated or mined from the literature and lack the breadth of data types present in Open Targets. To our knowledge, our results provide the first empirical evidence that the data hosted on the platform can provide real insights into target discovery, rather than just being a collection of genes with disease associations.

We acknowledge a potential limitation of our approach is the labelling of the classes. First, we do not have a pure negative class, which makes a binary classification task much more challenging than in a conventional supervised setting: this is because defining *bona fide* unsuccessful targets is extremely difficult, if possible at all. When selecting our positive set of targets using the Informa Pharmaprojects data [32], we ignored a number of categories relating to drug programmes that had been suspended or discontinued, as well as drugs that were withdrawn from market. However, it is often unclear what the reason for failed or abandoned programmes is; for example, there could be perfectly viable targets whose drugs have been withdrawn from the market for commercial reasons, or whose programmes have been halted because of changing R&D strategies. Moreover, a target that is unsuccessful for a particular indication could eventually be successful in a different therapeutic area. Second, using an unlabelled set containing both negatives and potential positives will invariably affect most of the performance measures that are traditionally used in standard supervised scenarios. Specifically, the number of true positives is going to be underestimated [48], which leads to worse precision and sensitivity/recall estimates. The latter was found to be considerably lower compared to other metrics in our case, and we believe this is due at least in part to the underestimation of true positives that occurs when noisy negative labels are used. Finally, we defined our positive set from drugs that are currently on the market or under active development in the pharmaceutical industry. In other words, we have taken a snapshot of the therapeutic targets we know are working at the moment. However, not all of the targets that are currently being worked on will be successful (i.e.: they will end up with a marketed drug); a number of these are indeed destined to fail, which makes our positive set not immune from potential misannotations either. Despite these caveats we believe the annotation strategy we used best describes the current target landscape in the pharmaceutical industry and makes it possible to employ an effective semi-supervised learning approach. Finally, in terms of performance, a model with 71% accuracy is certainly less than ideal. However, it is important to emphasise that such an approach could already have a significant impact in drug development considering the current success rates across the pharmaceutical industry [3, 4].

As the size and quality of datasets and the power of computing infrastructures increase, applications of machine learning are becoming more and more successful in biology [55, 56] and genomics [57], and we expect more refined machine learning paradigms and models coupled with more comprehensive datasets to achieve substantially better performance than the models presented here. In particular, both the fields of computational biology and drug discovery might be poised for a deep learning revolution [58–61] and a more sophisticated architecture of our feed-forward neural network algorithm with additional hidden layers could already show important performance gains. Future developments of the Open Targets platform, with more data and potentially more data types added could also improve the performance significantly. Notably, in an effort to assess the predictive power of gene–disease association data, we limited ourselves to the data available in the Open Targets platform. Inclusion of functional (Gene Ontology, pathways), structural (protein domains) or interaction data (protein–protein interactions) is likely to have a large impact on the ability to successfully predict therapeutic targets.

Indeed, these data types have already been reported to be good predictors of druggability (intended as the ability of a protein to bind to a compound that alters its activity with a therapeutic effect), with models achieving AUC measures between 0.69 and 0.93, depending on the approach and data types utilised [62–70]. Similarly, gene–disease association data has been used before to discover new genes with important roles in disease, with precision estimates ranging from 0.61 to 0.84 [64, 71–73]. Interestingly, this is another scenario where sourcing unambiguous negative examples is challenging and has often been framed as a PU learning problem [71–73].

Conversely, our objective was to demonstrate that disease association data can predict therapeutic targets: to our knowledge, this is the first study of its kind. It is important to stress that we don’t make any claim regarding the druggability of these targets: we expect them to have an interesting disease association profile similar to that of existing drug targets, but they may well be currently undruggable. Besides, the concept of druggability is likely to change over the years as newer technologies such as RNAi [74] and CRISPR/Cas9 [75] emerge and realise their potential in drug discovery.

While this study focuses on prediction, we haven’t neglected inference. Despite the fact that the four algorithms we evaluated are well known to be black boxes, we utilised a feature importance workflow and observed the classification criteria of a simple decision tree to gain some insights into what are the characteristics of therapeutic targets that are currently being successful.

One of the strengths of the Open Targets platform is that it features unbiased genome-wide data for a number of its data types (germline and somatic DNA mutations, RNA expression). Importantly, the animal model data type is a low throughput approach that is likely to be biased towards well studied genes, diseases or phenotypes. Arguably, there are more animal models for genes that can potentially be therapeutically targeted or are currently being progressed as targets, compared to the rest of the genome. We believe this can explain, at least in part, why the animal model feature in the Open Targets data appears to have rich information content for discriminating therapeutic targets in our models. Although animal models are not always going to adequately translate and be relevant to drug discovery in humans [76], our analysis suggests that data on mutant mice exhibiting human disease-relevant phenotypes can predict therapeutic targets. This is particularly relevant in light of the fact that 40% of programme failures for efficacy reasons are due to either poor linkage between target and disease or absence of good animal models for the disease under investigation [4]. Whether the influence of animal model data on drug discovery programmes will continue as evidence from primary human cells and populations is prioritised (or as more mouse knockouts are developed) remains to be seen.

Gene expression data has been utilised broadly in drug discovery and development to gain better understanding of pathological conditions as well as to test the effect of compounds at a genome-wide scale [77, 78]. The connectivity map approach for drug repositioning for example, relies entirely on gene expression data [79]. Our findings confirm that altered RNA expression in diseased tissue is a key data type defining therapeutic targets that are currently on the market or being explored. Similarly, the proportion of targets with human genetic evidence was recently reported to increase significantly across the drug discovery pipeline and it was proposed that progressing targets with genetic links to the disease under investigation could double clinical success rates [7]. The results presented here are in line with these observations as genetic association was found to be one of the defining characteristics of current and established targets in the pharmaceutical industry.

As for the remaining data types we investigated, the presence of a gene in a pathway that is altered in disease is likely to be relevant, but we are aware that the affected pathway data type is currently much less represented in the Open Targets platform compared to other data types. We also note that somatic mutations are extremely significant for oncology indications but their contribution is probably diluted when collapsing all evidence at the gene level by averaging across indications.

And indeed, we do expect contributions from different data types to vary, even considerably, across different disease areas. It is plausible that some data types could be highly predictive of current drug targets in a particular disease and be poor predictors in another area. In line with this, genetic support varies significantly across indications, with metabolic and digestive diseases being examples of therapeutic areas having high and low levels of genetic associations with drug targets, respectively [7]. We attempted to run our predictive workflow on the same data independently on each therapeutic area but failed to produce models with decent performance, probably because of the reduced number of observations (data not shown).

Of the known targets we made predictions for, our model correctly classified later stage drug targets more easily than earlier stages targets. We believe this reflects the fact that targets more advanced in the pipeline will have more established links with the disease area of interest and are therefore more straightforward to tease apart by a classifier. This is extremely clear for targets with launched drugs that invariably exhibit a very strong profile of disease association, something that should be used as an imperative guideline as we aim to progress new targets across the pipeline. The greater uncertainty of our model in classifying preclinical and early clinical data can be attributed to the fact that these targets have lower levels of disease associations overall and that indeed some of them will fail as they progress through the pipeline. We also note that our model predicts as non-targets targets associated with drug programmes discontinued during development much more often than it predicts them as targets, thus reinforcing the notion that putative targets with poor disease linkage are more likely to fail in the drug discovery pipeline.

We carried out predictions on more than 15,000 proteins that are not currently being pursued by pharmaceutical companies (Additional file 5: Table S1). Since we only label as positives drug targets that are currently on the market or under active investigation, there is a chance that we will predict targets that have extensively been explored by pharmaceutical companies and have failed. This is indeed the case with some of our top hits in the prediction set, such as metalloproteinases (e.g.: MMP3, MMP7, MMP10, MMP13, MMP14, MMP20). These proteins have been thoroughly examined as drug targets, predominantly in cancer [80] and arthritis [81]. While most clinical programmes failed due to lack of specificity or incomplete understanding of the disease biology, there is renewed interest in this family of proteins as disease understanding improves significantly and new pharmaceutical technologies emerge [82, 83]. Our results confirm that several metalloproteinases have indeed an attractive profile of disease association, presumably very similar to that of more established therapeutic targets. Metalloproteinases, and similar targets without current active programmes, could be of particular interest from a drug repositioning perspective. Combining good druggability and promising disease association profiles, these abandoned targets could be tested in new therapeutic areas where compelling evidence exists. Another high-scoring hit, BRWD1 is a putative chromatin remodelling protein that belongs to the bromodomain family [84, 85] and has a role in cytoskeleton organisation [86]. Despite a poor functional annotation, multiple mouse models clearly linking it to a number of reproductive system conditions exist [87, 88]. Of note, bromodomain-containing epigenetic regulators are well-studied drug targets across different therapeutic areas [89]. We identify several others genes that we believe have a potential to become therapeutic targets in the future: RAB18 is a small GTPase that could be targeted to halt or reduce dengue virus infection [90]; blockers of KCNB1, a voltage-gated ion channel, have been suggested as hypoglycaemic agents for type II diabetes [91–93]; TAB 1, a TGFB downstream effector that activates the MAP kinase TAK1 [94], has been validated as a triptolide target in macrophages and has promise as a therapeutic target for immunoinflammatory indications [95].

## Conclusions

In summary, we exploited the notion that poor linkage between targets and diseases correlates with clinical failure to build a machine learning framework able to make accurate predictions of therapeutic targets, exclusively using gene–disease association data. Our predictions can be considered for further analysis by the wider target discovery community, whilst remaining mindful that true and complete target validation only occurs when drugs show efficacy and safety profiles that allow them to be marketed and used by patients. We believe that, as an industry, we need to focus on clear-cut and unambiguous evidence linking genes and diseases to maximise our chances of success; in particular, animal model, genetic and gene expression evidence should be among the data types driving the target discovery process. Finally, we welcome initiatives aimed at the comprehensive annotation of gene–disease relationships such as Open Targets that have a real potential to catalyse a more forward-looking and data-driven target discovery process in the years ahead.

## Additional files

**Additional file 1: Figure S1.** Exploratory data analysis of the working dataset. (A) Hierarchical clustering using Euclidean distance and Ward’s linkage: columns represent features, rows represent genes and are coloured according to their label (green: target; purple: non-target); (B) Principal Component Analysis: each dot represents a gene and is coloured according to its label (green: target, purple: non-target).

**Additional file 2: Figure S2.** Estimated performance measures of trained classifiers as assessed by nested cross-validation on the training set. (A) Precision–recall curves; (B) Box plot showing estimated misclassification errors for the four algorithms, as assessed by nested cross-validation on the training set.

**Additional file 3: Figure S3.** Overlap of predictions across classifiers. Venn diagrams showing the relative overlap of (A) predicted targets and (B) predicted non-targets for the four algorithms as evaluated by nested cross-validation on the training set.

**Additional file 4: Figure S4.** Monte Carlo simulation to assess the effect of randomly sampling from the unlabelled class on the classifier performance. Ten thousands random samples of the unlabelled class were aggregated to the positive class and used to train and test a NN classifier. Histograms show distributions of (A) accuracy (mean = 0.71, standard deviation = 0.02) and (B) AUC (mean = 0.77, standard deviation = 0.02) calculated using the test set.

**Additional file 5: Table S1.** Prediction results of neural network classifier. The first three columns contain gene identifiers, the fourth column is the prediction (0: non-target, 1: target), the fifth and sixth columns contain the predicted probabilities of being a target or not, respectively.

**Additional file 6: Table S2.** Literature text mining results. The first two columns contain gene identifiers, the third column contains the PubMed ID of the publication where the gene is mentioned as a therapeutic target.

**Additional file 7: Figure S5.** Permutation test to assess the significance of the literature-based validation. Ten thousands permutations of the Fisher’s exact test were run using random labels. Histograms show distributions of (A) p values (mean = 0.41, standard deviation = 0.42) and (B) odds ratios (mean = 1.00, standard deviation = 0.08).

## Abbreviations

AUC: area under the curve
FPR: false positive rate
GBM: gradient boosting machine
GWAS: genome-wide association study
NN: neural network
PU: positive unlabelled
RF: random forest
ROC: receiver operating characteristic
SVM: support vector machine
t-SNE: t-distributed stochastic neighbour embedding
