# Supplementary figures and images for: In silico prediction of novel therapeutic targets using gene–disease association data
Source: J Transl Med. 2017 Aug 29;15:182. doi: 10.1186/s12967-017-1285-6 (PMC5576250; doi:10.1186/s12967-017-1285-6)

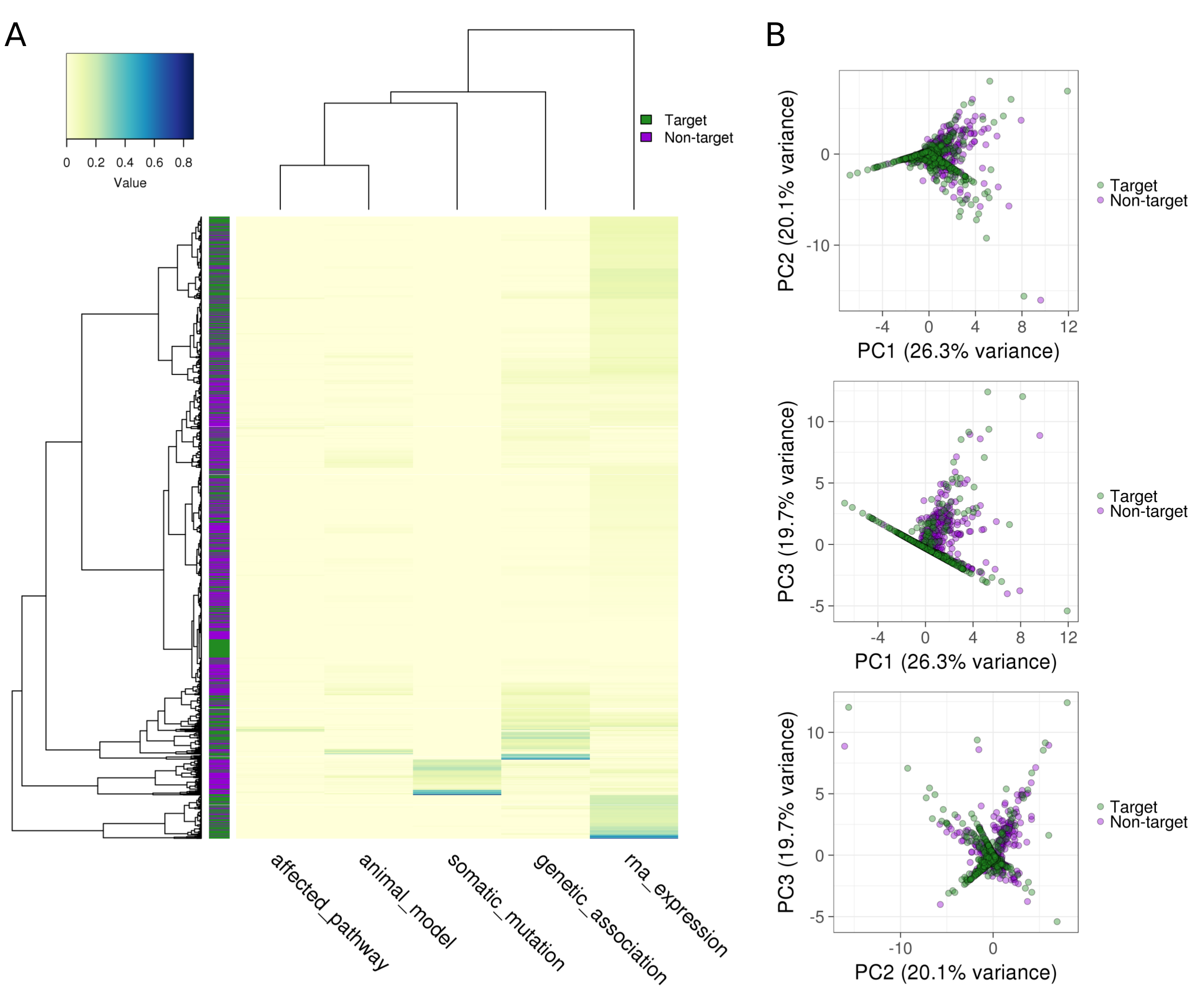

Supplement: Supplementary file 1 — Additional file 1: Figure S1. Exploratory data analysis of the working dataset. (A) Hierarchical clustering using Euclidean distance and Ward’s linkage: columns represent features, rows represent genes and are coloured according to their label (green: target; purple: non-target); (B) Principal Component Analysis: each dot represents a gene and is coloured according to its label (green: target, purple: non-target). [file 12967_2017_1285_MOESM1_ESM.tiff]

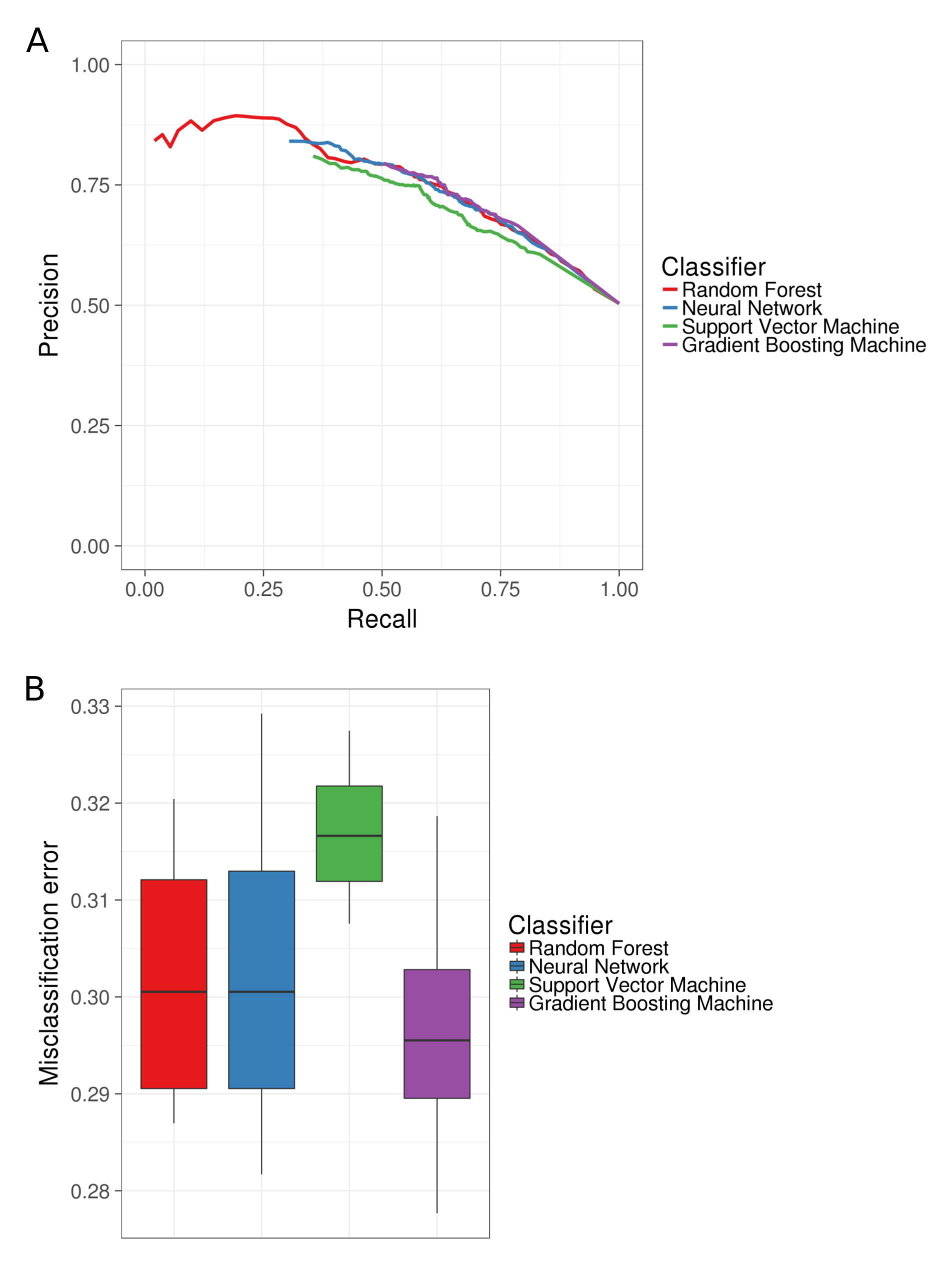

Supplement: Supplementary file 2 — Additional file 2: Figure S2. Estimated performance measures of trained classifiers as assessed by nested cross-validation on the training set. (A) Precision–recall curves; (B) Box plot showing estimated misclassification errors for the four algorithms, as assessed by nested cross-validation on the training set. [file 12967_2017_1285_MOESM2_ESM.tiff]

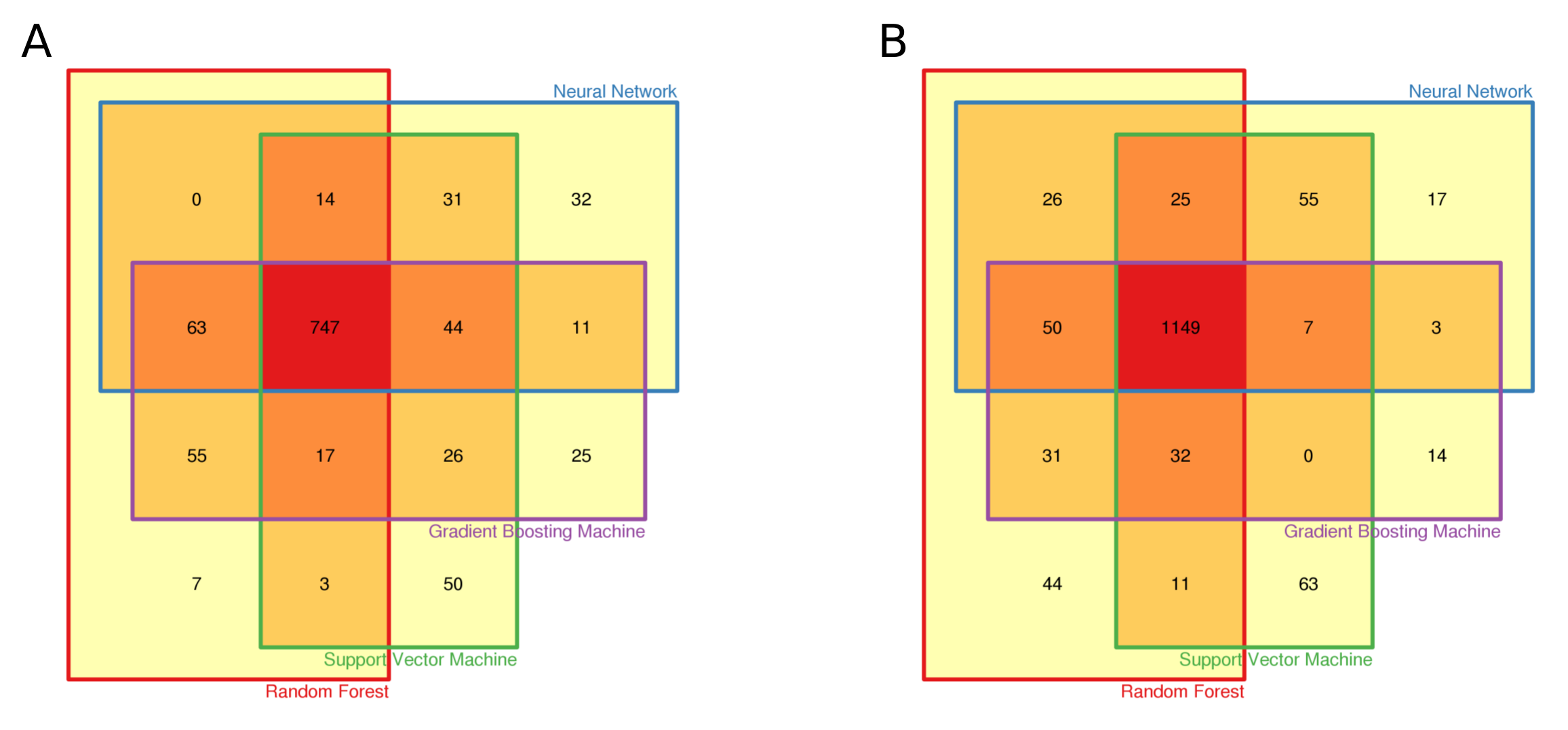

Supplement: Supplementary file 3 — Additional file 3: Figure S3. Overlap of predictions across classifiers. Venn diagrams showing the relative overlap of (A) predicted targets and (B) predicted non-targets for the four algorithms as evaluated by nested cross-validation on the training set. [file 12967_2017_1285_MOESM3_ESM.tiff]

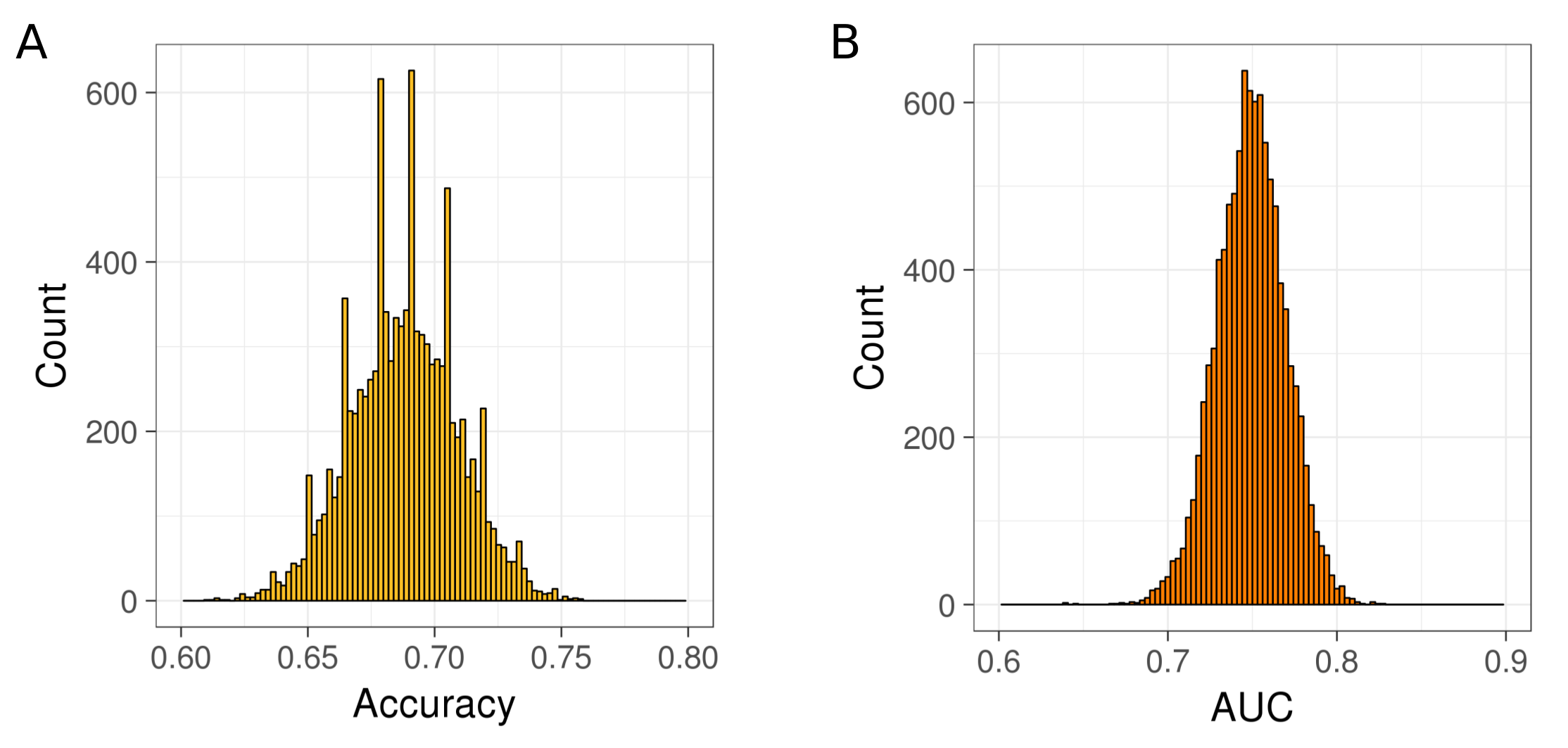

Supplement: Supplementary file 4 — Additional file 4: Figure S4. Monte Carlo simulation to assess the effect of randomly sampling from the unlabelled class on the classifier performance. Ten thousands random samples of the unlabelled class were aggregated to the positive class and used to train and test a NN classifier. Histograms show distributions of (A) accuracy (mean = 0.71, standard deviation = 0.02) and (B) AUC (mean = 0.77, standard deviation = 0.02) calculated using the test set. [file 12967_2017_1285_MOESM4_ESM.tiff]

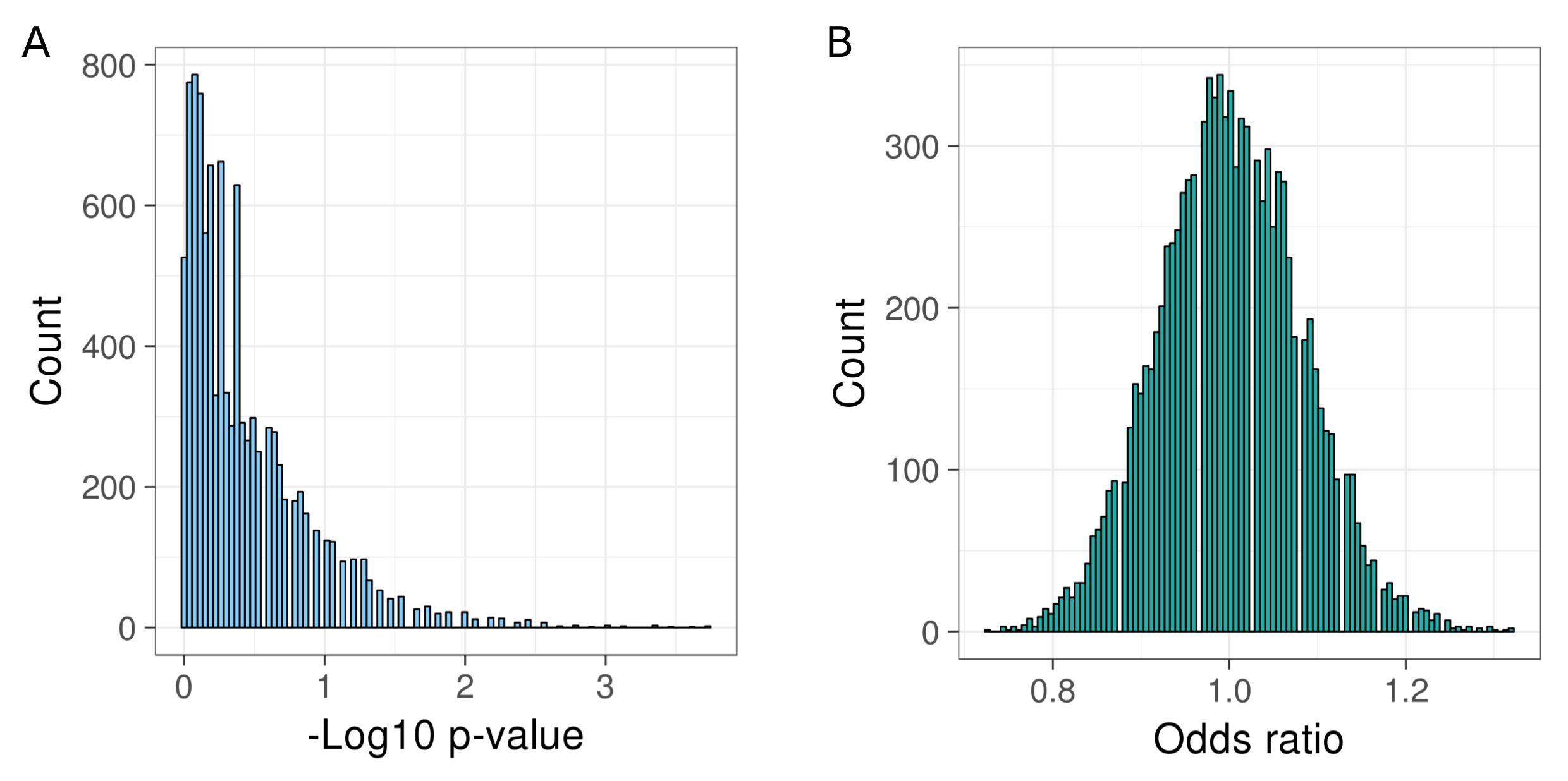

Supplement: Supplementary file 7 — Additional file 7: Figure S5. Permutation test to assess the significance of the literature-based validation. Ten thousands permutations of the Fisher’s exact test were run using random labels. Histograms show distributions of (A) p values (mean = 0.41, standard deviation = 0.42) and (B) odds ratios (mean = 1.00, standard deviation = 0.08). [file 12967_2017_1285_MOESM7_ESM.tiff]
